# Tracking the connection between evolutionary and functional shifts using the fungal lipase/feruloyl esterase A family

**DOI:** 10.1186/1471-2148-6-92

**Published:** 2006-11-08

**Authors:** Anthony Levasseur, Philippe Gouret, Laurence Lesage-Meessen, Michèle Asther, Marcel Asther, Eric Record, Pierre Pontarotti

**Affiliations:** 1 Phylogenomics Laboratory. EA 3781 Evolution Biologique Université de Provence, Case 19, Pl. V. Hugo, 13331 Marseille Cedex 03, France; 2 UMR 1163 INRA de Biotechnologie des Champignons Filamenteux, IFR86-BAIM, Universités de Provence et de la Méditerranée, ESIL, 163 avenue de Luminy, Case Postale 925, 13288 Marseille Cedex 09, France

## Abstract

**Background:**

There have been many claims of adaptive molecular evolution, but what role does positive selection play in functional divergence? The aim of this study was to test the relationship between evolutionary and functional shifts with special emphasis on the role of the environment. For this purpose, we studied the fungal lipase/feruloyl esterase A family, whose functional diversification makes it a very promising candidate.

**Results:**

The results suggested functional shift following a duplication event where neofunctionalisation of feruloyl esterase A had occurred with conservation of the ancestral lipase function. Evolutionary shift was detected using the branch-site model for testing positive selection on individual codons along specific lineages. Positively selected amino acids were detected. Furthermore, biological data obtained from site-directed mutagenesis experiments clearly demonstrated that certain amino acids under positive selection were involved in the functional shift. We reassessed evolutionary history in terms of environmental response, and hypothesized that environmental changes such as colonisation by terrestrial plants might have driven adaptation by functional diversification in Euascomycetes (*Aspergilli*), thus conferring a selective advantage on this group.

**Conclusion:**

The results reported here illustrate a rare example of connection between fundamental events in molecular evolution. We demonstrated an unequivocal connection between evolutionary and functional shifts, which led us to conclude that these events were probably linked to environmental change.

## Background

During evolution, new functions are acquired as a consequence of an accumulation of substitutions that alters the coding or regulatory sequences of genes. These evolutionary events are a key factor driving functional shift at transcriptional or biochemical level as well as shifts in sub-cellular localisation. There may be a range of knock-on effects on descent, ranging from no phenotypic novelty to whole new developmental processes such as those triggered by the cascades of gene expression in the case of neo-expression of a master regulator gene [1]. It is a major challenge to establish whether these mutations are fixed into a population by neutral genetic drift or via positive selection, and thereby evaluate the impact of the environment on this fixation. However, the literature reports very few unambiguous connections between changes in amino acid substitution rate (evolutionary shift) and functional shifts, and the role played by the environment is often limited to speculative discussion. Depending authors suggest different criteria are required for establishing a relationship, thus leading to different levels of confidence. Fundamental criteria for studying molecular adaptation during protein evolution would consist in *i*) detecting evolutionary and functional shifts, *ii*) linking the sites under positive selection to the functional changes, *iii*) reassessing these evolutionary events in terms of response to environmental context, *iv*) studying whether the novelty confers a selective environmental advantage, and v) studying whether functional convergence occurred under the same environmental changes. At present, the most commonly cited examples of adaptation involve the relationships between particular phenotypic shifts in response to a given environmental change, and many of these studies either did not conduct evolutionary analysis or failed to detect positive selection [2,3]. It is important to confirm that the sites identified as being under positive selection are actually involved in the functional novelty before a clear positive selection-driven connection can be established between evolutionary and functional shifts. Thereafter, the next step is to identify the impact of environment context in these events and establish whether the functional novelty responds to the environmental change and confers a selective advantage for the species. To our knowledge, only a few examples of functional gain fulfil all these criteria. The best studies stem from human artificial selection (antibiotic, herbicide, and insecticide resistance genes) where resistance to drugs is essential for survival [4,5]. To date, the full set of criteria listed above has only been demonstrated for artificial selections such as drug resistance and for one natural case concerning the digestive RNases in Asian and African leaf monkeys [6]. The case of RNaseI from ruminants also offers a strong example, with the novel biochemical function (i.e. the ability to digest single-stranded RNA at low pH) attributed to positively-selected sites [7]. This functional shift probably occurred in response to the acquisition of herbivory which conferred an advantage for ruminants eating grass. The examples of the retinal-binding membrane protein proteorhodopsin in marine bacteria, where positively-selected sites play a critical role in spectral light absorption, should also be highlighted since it illustrates how the distribution of different λ max is correlated to harvest depth, thus confirming an environmental link [8]. Finally, there are other examples where positively-selected sites are involved in the functional shift but without there being a clear link with the environmental shift [9-12].

Evolutionary shift is usually detected by estimating the rates of synonymous and nonsynonymous substitutions (ω = *d*_N_/*d*_S_) with ω = 1, < 1 and > 1 indicating neutral evolution, purifying selection and positive selection, respectively [13,14]. Given the strong selective pressure they are subjected to, many amino acids are not replaced, and adaptive evolution only operates at a few sites over a relatively short evolutionary period. A model that allows ω ratio to vary among sites and lineages was therefore developed to improve the detection of positive selection [13,15,16]. This model is specifically designed to analyse the evolution of gene families where functional divergence may have caused adaptive evolution. The detection of functional shift often hinges on preliminary experimental assays. However, there are databases containing experimental records that be retrieved automatically and then used to search for and detect functional divergence in large-scale studies of multigenic families.

In the present study, we tested the robustness of the connections between evolutionary and functional shifts, focussing specifically on the role of the environment. The objective was to conduct a comprehensive analysis of the multigenic families in which functional divergence has been detected, and to test the relationships between evolutionary, functional and environmental shifts. In order to test these connections, we first focused on an analysis of the well-described fungal lipase/feruloyl esterase A family. Type-A feruloyl esterases (E.C. 3.1.1.73) are enzymes responsible for cleaving the ester link between the polysaccharide main chain of xylans or pectins and monomeric or dimeric ferulic acid. Thus, feruloyl esterases make the cell wall increasingly vulnerable to further enzymatic attack [17]. A previous study, proposed a functional classification of the feruloyl esterases with four subclasses that were characterised and termed type-A, B, C and D [18]. However, lipases (E.C. 3.1.1.3) catalyse both the hydrolysis and the synthesis of esters formed from glycerol and long-chain fatty acids [19]. Despite strong structural and sequence similarities, this family presents two distinct enzymatic activities, i.e. lipase and type-A feruloyl esterase, that may have originated from evolutionary events such as functional shift.

The evolutionary history of this family was reconstructed, and the branch-site model was applied to test for positive selection. We tested the connection between evolutionary and functional shifts and then reassessed the connection in terms of response to environmental context..

## Results

### Evolutionary analysis of the homologous proteins from the lipase/feruloyl esterase A family

**Figure 1:**
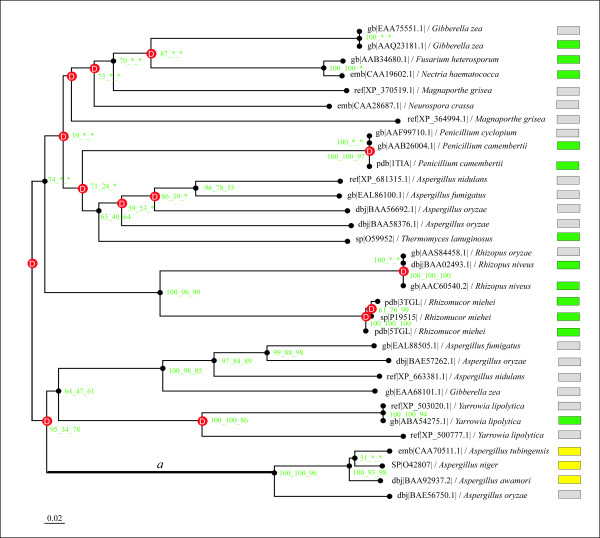
**Phylogenetic tree of the lipase/feruloyl esterase family**. This phylogenetic tree corresponds to the fusion of the three trees independently reconstructed using Neighbour Joining, Maximum Parsimony and Maximum likelihood methods. The green, yellow and grey boxes symbolise lipases, (type-A) feruloyl esterases (experimental data) and hypothetical proteins, respectively. The scale-bar represents the number of changes per position per unit of branch length. Red boxes represent gene duplication events. The bootstrap values (green numbers at nodes) are given for the three methods when the three trees share an identical node (NJ, MP, ML, respectively). Branch *a *is considered as the foreground branch for branch-site models.

All homologous proteins of the FAEA from *A. niger *were included in a phylogenetic analysis, and the corresponding phylogenetic tree was constructed (Fig. 1). Homology relationships between fungal lipases and feruloyl esterases (type A) assume a shift in at least one of these enzyme categories that led to a functional novelty in the fungi kingdom.

Phylogenetic analysis identified a monophyletic group containing both the feruloyl esterase A and lipase activities, whereas to date only lipase activity has been found in a paraphyletic group. Therefore, lipases currently appear to be the primitive function while the feruloyl esterase A could be the derived character. Feruloyl esterases A appeared following a duplication event that occurred before the separation of the *Aspergilli *(Euascomycetes) and yeasts (Hemiascomycetes). The robustness of this duplication node was confirmed by all three methods of phylogenetic tree reconstruction, i.e. Maximum Parsimony, Neighbor Joining and Maximum Likelihood methods, with high bootstrap values of 95 and 78 for the Neighbor Joining and Maximum Likelihood methods, respectively. Given the duplication node and the appearance of the derived character, we hypothesised that positive selection occurred along the lineage ancestral to *Aspergilli-*feruloyl esterase, and the branch was labelled as *a*. To test evolutionary shift using Maximum Likelihood analysis, we applied the branch-site model A developed by Yang and Nielsen [15] to the dataset, with branch *a *considered as the foreground branch and all others as background branches in the gene phylogeny. Parameter estimates under model A suggested that 45% of sites were highly conserved across all lineages with ω_0 _= 0, whereas 17% of sites were identified under the neutrality assumption with ω_1 _= 1 fixed and 37% of sites were under strong positive selection with ω_2 _= 999 (Table 1). This model was compared to the following null models: model M1a (neutral) for test 1 and model A but with ω_2 _= 1 fixed for test 2 (Table 2). The likelihood ratio tests (LRTs) gave 2Δ*l *= 53.78 with *P *< 10^-4 ^and df = 2 for test 1 and 2Δ*l *= 28.22 with *P *< 10^-4 ^and df = 1 for test 2. Furthermore, similar results (summarized in Additional File 1) were obtained using the branch-site Model B. These tests provided highly significant evidence of the presence of sites under positive selection. The Bayes empirical Bayes (BEB) analysis identified 33 sites under positive selection along branch *a *at the high probability level of > 95%. The vast majority (31 out of 33) of these positively selected sites are conserved in the feruloyl esterase A group but not in the lipase group. This pattern is typical of type I functional divergence [20]. Therefore, purifying selection had occurred after the positive selection period, thus confirming the important functional role of these sites for feruloyl esterase A activity. In order to test the sensitivity and robustness of these results, we conducted two LRTs under two different assumptions on codon usage: *i*) the Fequal model, which assumes equal codon frequencies and ignores codon usage bias, and *ii*) the Fcodon model that considers all 61 codon frequencies as parameters. These controls are useful because codon usage bias can have radical effects on the estimation of ω. Despite small variations, the LRTs were highly significant, thus providing further evidence of the presence of sites under positive selection among the foreground branch (Table 2). Furthermore, among the sites previously confirmed as positively selected, all (F3x4) were identified by BEB analysis using the Fcodon model and 15 were detected using the Fequal model (see Additional File 2). Our conclusion on the estimation of ω was therefore robust to assumptions on codon usage bias.

**Table 1:** Parameter estimates for the lipase/esterase data (n = 29)

Model	*p*	*l*	Parameters estimates	Positively selected sites
M0: one-ratio	1	-13903.33	ω = 0.0685	None
				
Branch-site model:				
Model A	3	-13792.38	*p*_0 _= 0.452, *p*_1 _= 0.170 (p_2 _+ *p*_3_) = 0.376 ω_2 _= **999**	Site for foreground lineage: 4Q 13R 17M 19T 22Q 26A 29C 40K 42Y 51W 53L 63T 69G 71D 75Q 76L 78T 80Y 100Y 103G 112E 137S 142T 145Q 147S 163S 195G 198N 204E 215S 236E 238Q 244N (at *P *> 0.95)

Note: *p *is the number of free parameters for the ω ratios. Parameters indicating positive selection are presented in boldtype. Sites potentially under positive selection were identified using the mature FaeA (*A. niger*) according to the Bayes empirical Bayes analyses for Model A. Note that the result of ω_2 _= **999 **corresponded to a boundary of the estimated parameter value, since dS was zero for this branch.

**Table 2:** The effects of codon usage bias on LRTs (n = 29)

		**Model**		
		
		**F equal**	**F3×4**	**F codon**
Estimates under model A		*p*_0 _= 0.427, *p*_1 _= 0.241 (p_2 _+ *p*_3_) = 0.330 ω_2 _= 11.182	*p*_0 _= 0.452, *p*_1 _= 0.170 (p_2 _+ *p*_3_) = 0.376 ω_2 _= 999	*p*_0 _= 0.469, *p*_1 _= 0.157 (p_2 _+ *p*_3_) = 0.372 ω_2 _= 999
				
Estimates under M1a		*p*_0 _= 0.627, *p*_1 _= 0.372 ω_0 _= 0.193	*p*_0 _= 0.711, *p*_1 _= 0.288 ω_0 _= 0.126	*p*_0 _= 0.730, *p*_1 _= 0.269 ω_0 _= 0.113
				
Estimates under model A (ω_2 _= 1)		*p*_0 _= 0.437, *p*_1 _= 0.259 (p_2 _+ *p*_3_) = 0.302 ω_0 _= 0.187	*p*_0 _= 0.480, *p*_1 _= 0.189 (p_2 _+ *p*_3_) = 0.329 ω_0 _= 0.121	*p*_0 _= 0.505, *p*_1 _= 0.178 (p_2 _+ *p*_3_) = 0.316 ω_0 _= 0.107
		
2Δ*l*	*Test 1*:	35.44	53.78	54.38
	*Test 2*:	19.98	28.22	27.89

Note: Branch-site model A was compared to site model M1a and model A with ω_2 _fixed to 1 for test 1 and 2, respectively. All tests are significant at 1%; with df = 2 and df = 1 for tests 1 and 2, respectively.

In order to test whether codon usage bias is lineage-specific (between feruloyl esterase A and lipase group), we used ENC to measure average codon usage bias. The averages measured for the feruloyl esterase A and lipases groups were 52.5 and 47.2, respectively, indicating that codon usage bias was not lineage-specific.

These results demonstrated that positive selection occurred along the branch leading to the feruloyl esterase A function in *Aspergilli*.

### Relationship between evolutionary and functional shifts

**Figure 2:**
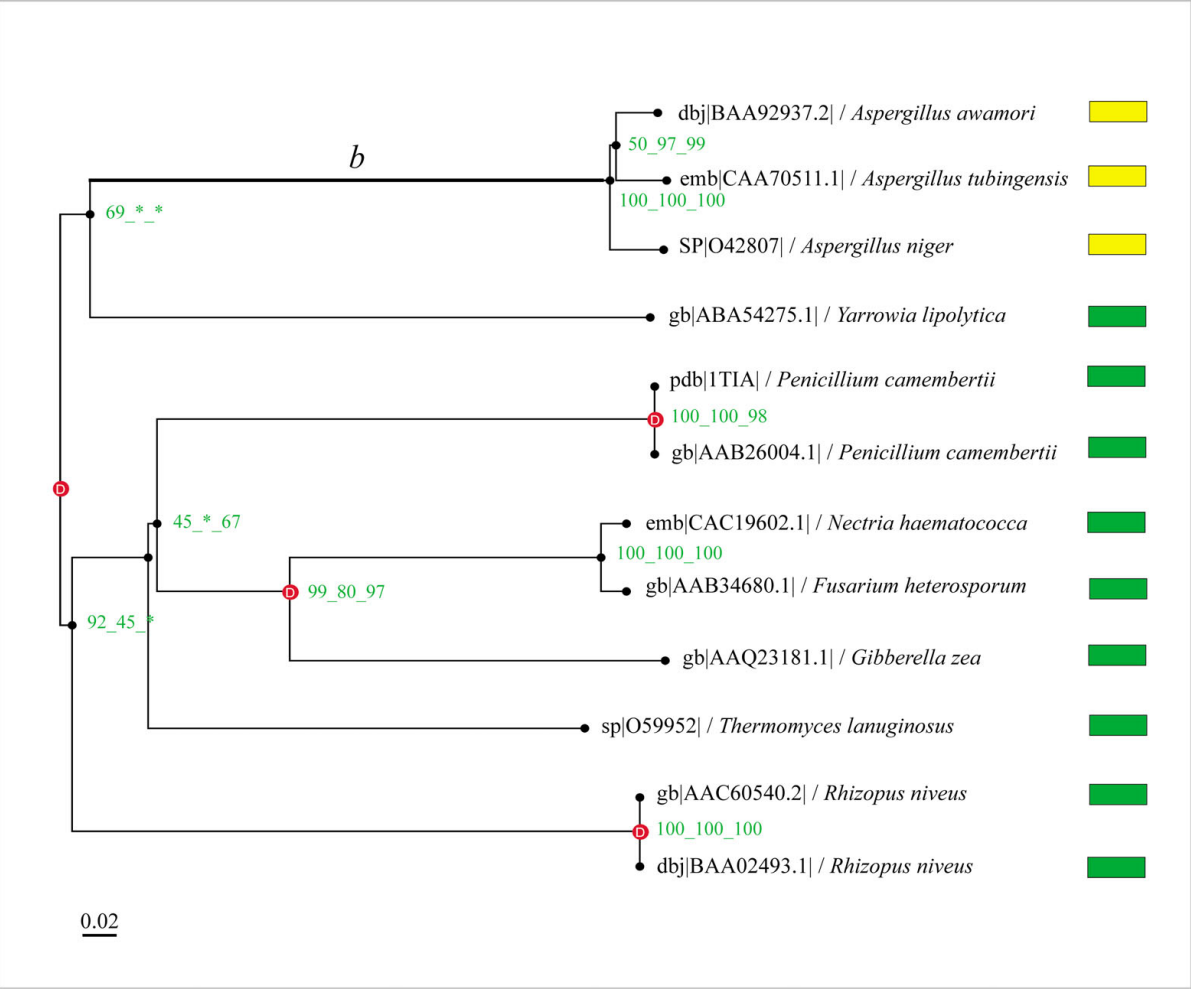
**Phylogenetic tree of the lipase/feruloyl esterase family (experimental data)**. This phylogenetic tree was constructed by integrating functionally described proteins. Green and yellow boxes show lipases and (type-A) feruloyl esterases (experimental data), respectively. The scale-bar represents the number of changes per position per unit of branch length. Red boxes represent gene duplication events. The bootstrap values (green numbers at nodes) are given for the three methods when the three trees share an identical node (NJ, MP, ML, respectively). Branch *b *is considered as the foreground branch for branch-site models.

In order to connect evolutionary shift to functional diversification in the lipase-feruloyl esterase A family, molecular adaptive evolution detection was performed by integrating functionally described proteins. The purpose of this analysis was to confirm the robustness of our results by only using protein-encoding genes whose functions had been experimentally described. Branch *b *corresponded to the post-duplication branch (equivalent of branch *a*) and was labelled as the foreground branch (Fig. 2). Branch-site model A applied to this new dataset (n = 12) confirmed that a significant proportion (39%) of sites along branch *b *were under positive selection with ω_2 _= 14.2 (Table 3). Moreover, the robustness of these results was also controlled under different assumptions on codon usage, and the resulting LRT statistics were highly significant for all models, thus providing further evidence of the presence of sites under positive selection (Table 4). These results were in full agreement with our initial analysis and thus established a correlation between evolutionary shift and the functional diversification, i.e. the lipase and feruloyl esterase A activities.

**Table 3:** Parameter estimates for the lipase/esterase data (n = 12)

Model	*p*	*l*	Parameters estimates	Positively selected sites
M0: one-ratio	1	-5974.07	ω = 0.0621	None
				
Branch-site model:				
Model A	3	-5925.68	*p*_0 _= 0.503, *p*_1 _= 0.102 (p_2 _+ *p*_3_) = 0.393 ω_2 _= **14.201**	Sites for foreground lineage: 17M 19T 22Q 29C 51W 63T 70G 71D 74L 75Q 76L 103G 126A 163S 209G 226V 248T 257A (at *P *> 0.95)

Note: *p *is the number of free parameters for the ω ratios. Parameters indicating positive selection are presented in boldtype. Sites potentially under positive selection were identified using the mature FaeA (*A. niger*) according to the Bayes Empirical Bayes analyses for Model A.

**Table 4:** The effects of codon usage bias on LRT (n = 12)

		**Model**		
		
		**F equal**	**F3x4**	**F codon**
Estimates under model A		*p*_0 _= 0.51693, *p*_1 _= 0.152 (p_2 _+ *p*_3_) = 0.330 ω_2 _= 11.182	*p*_0 _= 0.503, *p*_1 _= 0.102 (p_2 _+ *p*_3_) = 0.393 ω_2 _= 14.201	*p*_0 _= 0.477, *p*_1 _= 0.095 (p_2 _+ *p*_3_) = 0.426 ω_2 _= 999
				
Estimates under M1a		*p*_0 _= 0.732, *p*_1 _= 0.267 ω_0 _= 0.187	*p*_0 _= 0.793, *p*_1 _= 0.206 ω_0 _= 0.073	*p*_0 _= 0.794, *p*_1 _= 0.205 ω_0 _= 0.062
				
Estimates under model A (ω_2 _= 1)		*p*_0 _= 0.492, *p*_1 _= 0.159 (p_2 _+ *p*_3_) = 0.348 ω_0 _= 0.165	*p*_0 _= 0.537, *p*_1 _= 0.112 (p_2 _+ *p*_3_) = 0.350 ω_0 _= 0.073	*p*_0 _= 0.533, *p*_1 _= 0.112 (p_2 _+ *p*_3_) = 0.354 ω_0 _= 0.039
		
2Δ*l*	*Test 1*:	22.85	33.78	35.27
	*Test 2*:	10.31	6.84	6.83

Note: Branch-site model A was compared to site model M1a and model A with ω_2 _fixed to 1 for test 1 and 2, respectively. All tests are significant at 1%; with df = 2 and df = 1 for tests 1 and 2, respectively.

In order to test whether positive selection also occurred in different lineages along the phylogeny, others branches were labelled as foreground branches. The calculations are summarized in Additional File 3. LRTs gave no significant results with ω_2 _> 1 except for the branches leading to the lipases from *Yarrowia lipolytica *(ABA54275.1) and *Thermomyces lanuginosus *(O59952). In both these cases, the sites under positive selection were not involved in a functional shift as the lipase function was conserved. Furthermore, there was no overlap between the positively selected sites and the sites identified for branch *b*, thus confirming a divergent evolution between lipases and feruloyl esterases A. Therefore, positive selection had also occurred along different branches but only branch *b *led to neofunctionalisation.

In conclusion, both phylogenetic and evolutionary data suggested that the ancestral function (lipase) had shifted, with molecular adaptation leading to a novel enzyme (type-A feruloyl esterase).

### Are positively selected sites involved in the functional shift?

**Figure 3:**
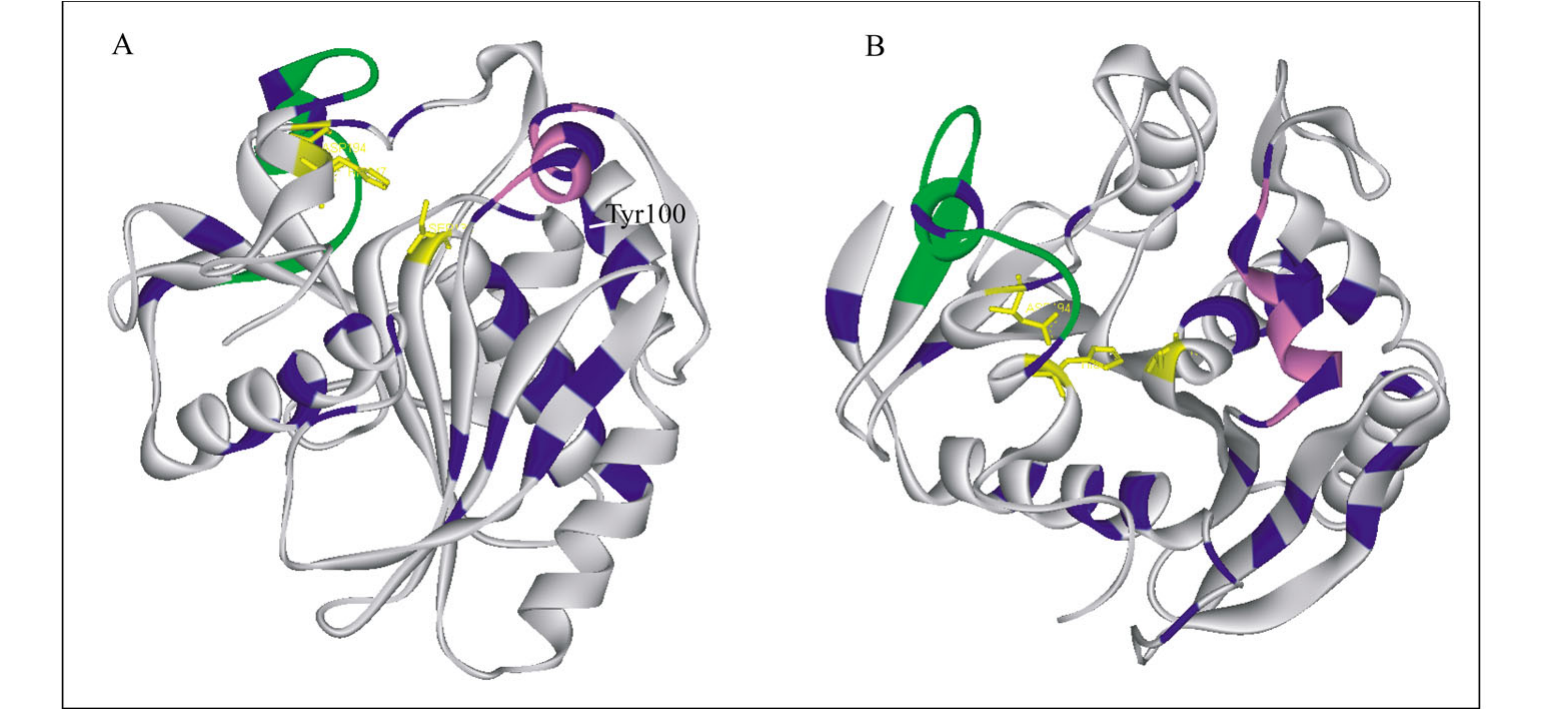
**Structural representation of AnFAEA**. The catalytic triad is displayed in yellow and the positively selected sites are coloured in blue. The flap region and the 226–244 loop are labelled in pink and green, respectively. Two different orientations (A and B) are depicted.

An essential prerequisite to connecting evolutionary and functional changes is to test whether positively selected sites are involved in the functional shift. As positively selected sites are assumed to be of major biological importance for type-A feruloyl esterases, we localised the 33 sites on the 3D structure of *A. niger *FAEA (Fig. 3) and integrated functional data from site-directed mutagenesis into our analysis (Table 5). Many positively selected amino acids were found in specific regions known to be of major importance for enzymatic activity and substrate specificity. This trend was particularly pronounced in the flap region of the AnFAEA (residues 69–80). A review of previous reports of mutational and structural analyses led to the identification of three clear areas, which are listed in table 5. The first is the flap region, which in AnFAEA exhibits a helical conformation similar to the open conformation of the lid found in lipases such as *Rhizomucor miehei*. The inner surface of the lid from *R. miehei *lipase is sufficiently hydrophobic to promote the association of the enzyme with a lipid interface, whereas the flap region of AnFAEA is more hydrophilic. Based on these results, three polar amino acids (Asp71, Gln75 and Thr78) located in the flap region were identified as being under positive selection, thus confirming the significance of the distinction (hydrophobic *versus *polar) between lipases and feruloyl esterases. Moreover, the important role of the flap residues was demonstrated by site-directed mutagenesis analyses, and the positively selected sites Asp71 and Tyr80 were shown to be determinant for catalysis and substrate discrimination. The role of Tyr80 was also underlined by other mutational analyses where k_cat _or substrate specificity was modified in response to the mutations designed at this site (Table 5). Secondly, it should be noted that Tyr100 was also a positively selected site. Indeed, given the close vicinity of catalytic serine residue and Tyr100, it could be argued that replacing a conserved Phe in the lipase family with Tyr100 in AnFAEA would reduce hydrophobicity at the bottom of the active sites, and could therefore affect substrate recognition. The mutation of the *Thermomyces lanuginose *lipase Phe100 to Tyr was demonstrated as an essential step in conferring ferulate ester-hydrolysing activity (Table 5). Taken together, these results confirmed that positively selected sites are unambiguously involved in the functional shift. Thirdly, the three positively selected sites (Glu236, Gln238, and Asn244) were all assumed to play a role in the difference in substrate specificity between lipases and type-A feruloyl esterases based on the structure of AnFAEA. Indeed, the active-site cavity is confined by the flap region (residues 69–80) and the 226–244 loop that exhibits structural plasticity for the substrate binding site. Thus, all three identified sites could play key roles in this essential region, especially in the 236–244 loop. In conclusion, biological data from mutagenesis experiments confirmed that the positively selected sites had been robustly identified, and made it possible to establish the connection between positively selected sites and functional shifts.

**Table 5:** Role of the positively selected sites in the discrepancy between feruloyl esterase A and lipase activities.

**Three dimensional localization**	**Positively selected sites**	**Site directed mutagenesis**	**& Role on Lip/FAE discrepancy**	**References**
1. Flap region (69–80)	71,75,78,80	80	catalysis and substrate discrimination	[45,46]
2. Catalytic vicinity	100	100	substrate recognition	[47]
3. Loop (226–244)	236,238,244	-	structured plasticity to the substrate binding site	[23,48]

## Discussion

Correlating evolutionary, functional and environmental shifts is a delicate task, and there are only a few published studies (described above) that establish a direct link between these shifts. The aim of this study was to extend our investigation into the relationship between evolutionary and functional shifts to focus on the role of the environment. For this purpose, we studied the fungal lipase/feruloyl esterase A family whose functional diversification makes it a promising candidate. One study showed that type-A feruloyl esterases were more sequence-related to lipases than they are to other groups (type B-D), but evolutionary analysis was not performed [18].

In this study, we constructed phylogenetic trees and identified positive selection along the branch leading to feruloyl esterase (type A) activity. Positively selected sites were identified using the Bayes empirical Bayes (BEB) procedure of the branch-site model A [15,21]. Indeed, BEB is a reliable method that gives a false-positive rate of less than 5% when sites are identified at the selected cut-off. As codon usage bias could have a stronger effect on estimation of ω than the transition/transversion rate bias, we estimated the effect of codon usage bias on LRTs [22]. The parameter estimates proved relatively stable, thus demonstrating that LRTs were robust to assumptions on codon usage bias. The next level of our analysis was to unambiguously demonstrate that the positively selected sites were indeed involved in the functional shift, which is a *sine qua non *condition to clearly establishing a connection between evolutionary and functional shifts. Based on site-directed mutagenesis data, we established that three sites under positive selection were involved in the functional shift leading to the type-A feruloyl esterase function. These mutational experiments made it possible to switch both enzymatic functions. Moreover, several positively selected amino acids were located in specific regions known to be of great functional significance for the substrate specificity and enzymatic activity of type-A feruloyl esterases and lipases. Hermoso *et al*. stated that active site plasticity between lipases and feruloyl esterase A depends on the nature of specific residues and on structural modifications within the active sites [23]. The 33 positively selected sites that we identified offer potential candidates for this plasticity. These results therefore made it possible to connect evolutionary and functional shifts.

Previous studies on site-directed mutagenesis focused on the replacement of target amino acids by comparing alignments between lipase and feruloyl esterase sequences and using 3D data to infer the potential biological importance of targeted sites.

For instance, a homologous position for a particular amino acid that is conserved in one subfamily but highly variable in another can be interpreted as functionally important in the first subfamily but less so in the second. In contrast, when radically different amino acids are fixed between subfamilies, the functional interpretation is that these sites fulfil different but equally important roles in the two subfamilies [24-26]. Positions with variable rates of divergence in different regions of a tree could provide a basis for relating individual amino acid residues to specific functional differences in certain branches [27,28]. Although these approaches, which are based solely on rate shifts, are implicitly linked to protein evolution and remain complementary to our analysis, they do not estimate the strength or direction of natural selection pressure. Here, we have provided an evolutionary dimension by identifying a branch containing a number of sites evolving very significantly faster than under neutrality. Moreover, these sites caused neofunctionalisation, meaning that functional shift is driven by positive selection.

The final step of our analysis focused on the second level of our investigation concerning the role of the environment in the adaptive evolution of the lipase/feruloyl esterase A family.; thus, evolutionary history was reconstructed and reassessed in terms of response to environmental pressure. There had been a duplication event occurring before the separation of the Hemiascomycetes (yeasts) and Euascomycetes (*Aspergilli*), and which was followed by a functional shift in the Euascomycetes but gene losses in the Hemiascomycetes. Functional diversification following gene duplication is a frequent event during evolutionary history of multigenic families. Here, phylogenetic and evolutionary data suggested that the ancestral function (lipase) was shifted, leading to enzymatic novelty (type-A feruloyl esterase). It is interesting to note that *Aspergilli *conserved both functions, i.e. the lipase and feruloyl esterase A activities, which makes it the only genus identified to date that possesses this distinctiveness. However, increasingly powerful genome sequencing capabilities coupled with biochemical experiments may lead to feruloyl esterase A activity being extended to other groups. Moreover, feruloyl esterase A activities could be functionally substituted by non-homologous proteins with similar enzymatic properties, for instance by the type-B feruloyl esterases.

Key insight to understanding the role of the environment would be gained by pinpointing the selective advantages that could be conferred by feruloyl esterase A activity. The *Aspergilli *genus is a well-known plant cell wall degrader, and feruloyl esterases are able to hydrolyse the ester bonds linking ferulic acid to plant cell wall polysaccharides. Thus, feruloyl esterases facilitated the access of main-chain-degrading enzymes to the polysaccharide backbone, thus allowing efficient lignocellulose degradation. The colonisation by the land plants (Embryophyta) that appeared during the Silurian period (-430 to -400 Myr) could have progressively driven neofunctionalisation in *Aspergilli*. This period estimation is strengthened by our phylogeny by the fact that the Hemiascomycete yeasts are believed to have diverged from a common ancestral fungus at least 400 million years ago. Enzymatic novelty in Euascomycetes could have generated a selective advantage that became fixed in this group during the emergence of the land plants. The appearance of feruloyl esterase A activities could be regarded as an efficient step among several possible routes towards plant colonisation. Therefore, we hypothesised that a connection between functional and environmental shifts could be established according to the impact of the role played by feruloyl esterase A in plant cell wall degradation. However, based on the knowledge currently available, this connection can only remain hypothetical.

Structural comparison between *Clostridium themocellum *feruloyl esterases (XynY and XynZ) and AnFAEA revealed that feruloyl esterases could have evolved through functional convergence following evolutionary divergence [23]. Future studies are needed to analyse whether convergence occurred in other enzymes involved in the same plant cell wall degradation functions as type-A feruloyl esterases. Indeed, the occurrence of convergence greatly strengthens the connection between evolutionary shift and environmental changes [5]. At present, several artificial conditions (e.g. antibiotic resistance genes) and only one natural case are able to identify unambiguous convergence via a clear connection between evolutionary (positively selected sites) and functional shifts [6]. It would be useful to study whether generalised environment-driven functional shifts could be have been responsible for enzymatic diversification in fungi kingdom.

The positively selected sites we identified could be used as "probes" for functional inference. These sites also represent potential novel targets in biotechnology for mutational analyses or in paleobiochemical experiments designed to resurrecting the ancestral function of the lipase/feruloyl esterase gene [29-31].

## Conclusion

In summary, these results illustrate a rare example of the connection between fundamental events of molecular evolution. We demonstrated that evolutionary shift (positively selected sites) has led to functional diversification that could be related to environmental changes. Future studies are scheduled to test other multigenic families in order to analyse and further characterise the general trends underpinning these connections.

## Methods

### Construction of phylogenetic trees

Phylogenetic analyses were performed using the automated genomic annotation platform FIGENIX [32] to retrieve sequences and alignments and perform phylogenetic reconstruction. The pipeline used applied three different methods of phylogenetic tree reconstruction, i.e. Maximum Parsimony [33], Maximum likelihood [34] and Neighbour Joining [35], and a midpoint-rooted consensus tree was built. Bootstrapping was carried out with 1000 replications. Bootstrap values are reported for each method (for a detailed description of the pipelines and models used, see [36]).

### Evolutionary analysis

#### Preparation of datasets

Protein and DNA sequences were retrieved from the National Center for Biotechnology Information [37]. The protein sequences were aligned using ClustalW [38]. Correspondence between protein alignment and each DNA sequence was established using the Wise2 software package followed by manual adjustments [39]. The final alignment contained 217 and 226 codons for dataset 1 (n = 29) and dataset 2 (n = 12), respectively.

#### Detection of positive selection

The codeml program of the PAML (Phylogenetic Analysis by Maximum Likelihood [13,40]) 3.15 software package was applied to test for positive selection. PAML uses a Maximum Likelihood algorithm to assign likelihood scores to different models for selection. If a higher likelihood score was obtained for a model incorporating positive selection than a null model without positive selection, this constitutes evidence for positive selection. We first used the model A implemented by Yang and Nielsen [41]. This model enables ω (= *d*_N_/*d*_S_) to vary both between sites and between lineages, and was implemented in the maximum likelihood framework. Branches *a *and *b *tested for positive selection were labelled as foreground branches, and all remaining branches were labelled as background branches. This model was then used to construct two likelihood ratio tests (LRTs) by comparison with a model that does not identify positive selection. The null hypothesis for test 1 is the site model M1a [21] which assumes two site classes with 0 < ω_0 _< 1 and ω_1 _= 1 for all branches. For test 2, the null hypothesis is the branch-site model A but with ω_2 _= 1 fixed. Positively selected sites were identified by the Bayes empirical Bayes (BEB) method [21]. The effect of codon usage bias on LRTs was estimated using two assumptions on codon usage: the Fequal model and Fcodon model.

#### Measurement of codon usage bias

Codon usage bias (effective number of codons [ENC]; [42]) was computed using DNASP software (version 4.10.9) [43].

#### Visualisation of protein structures

Protein structures were visualised using accelrys^® ^DS Visualizer 1.5, available at [44]. The PDB number of the FAEA from *A. niger *is 1USW.

## Abbreviations

AnFAEA, feruloyl esterase A from *Aspergillus niger*; Asn, asparagines; Asp, aspartic acid; BEB, Bayes empirical Bayes; ENC, effective number of codon; FAEA, type-A feruloyl esterase; Gln glutamine; Glu, glutamate; LRT, likelihood ratio test; NJ, Neighbor Joining; ML, Maximum likelihood; MP, Maximum Parsimony; PAML, Phylogenetic Analysis by Maximum Likelihood; Phe, phenylalanine; Thr, threonine; Tyr, tyrosine.

## Authors' contributions

AL designed and performed the whole analysis, interpreted the results and wrote the manuscript. PG supervised the phylogenetic studies with the creation of Figenix software. LLM and MA participated in results analysis. MA and ER participated in results analysis and supervised the study. PP supervised the whole study, participated in results analysis and helped draw conclusions. All the authors read and approved the final manuscript.

## Supplementary Material

Additional file 1 Table S1. Parameter estimates for the lipase/feruloyl esterase data under Model B and the effects of codon usage bias on LRTs (n = 29). Click here for file

Additional file 2 Table S2. Positively selected sites identified by Bayes empirical Bayes analysis using the Fcodon and Fequal models (n = 29). Click here for file

Additional file 3 Detection of positive selection along different branches in the gene phylogeny. All tests were performed as described for branch *b*. Click here for file
