# Supplementary material for: Tracking the connection between evolutionary and functional shifts using the fungal lipase/feruloyl esterase A family
Source: BMC Evol Biol. 2006 Nov 8;6:92. doi: 10.1186/1471-2148-6-92 (PMC1660568; doi:10.1186/1471-2148-6-92)
Supplement: Additional file 1 — Table S1. Parameter estimates for the lipase/feruloyl esterase data under Model B and the effects of codon usage bias on LRTs (n = 29). [file 1471-2148-6-92-S1.doc]

**Table S1. Parameter estimates for the lipase/feruloyl esterase data under Model B and the effects of codon usage bias on LRTs (n=29)**

Model *p* *l* Parameters estimates Positively selected sites

M0: one-ratio 1 -13903.33 ω = 0.0685 None

Branch-site model:

Model B 5 -13558.49 *p*_0_ = 0.355, *p*_1_ = 0.322 Sites for foreground lineage:

(p_2_ + *p*_3_ ) = 0.341 4Q 19T 22Q 26A 51W

ω_2_ = **11.06** 63T 75Q 76L 103G 147S

163T 236E (at *P* > 0.95)

Site-specific model:

M3 (*K*=2) 3 -13569.48 *p*_0_ = 0.49, *p*_1_ = 0.50 None

ω_0_ = 0.02, ω_1_ = 0.13

2∆*l* *Test 1*: 21.98

Note: *p* is the number of free parameters for the ω ratios. Parameters indicating positive selection are presented in boldtype. Sites potentially under positive selection are identified using the mature FaeA (*A. niger*) according to the Naive empirical Bayes of Model B. Test 1 comparing sites-specific model M3 and branch-site model B is significant at 1%; with df = 2.
