# Supplementary material for: Tracking the connection between evolutionary and functional shifts using the fungal lipase/feruloyl esterase A family
Source: BMC Evol Biol. 2006 Nov 8;6:92. doi: 10.1186/1471-2148-6-92 (PMC1660568; doi:10.1186/1471-2148-6-92)
Supplement: Additional file 2 — Table S2. Positively selected sites identified by Bayes empirical Bayes analysis using the Fcodon and Fequal models (n = 29). [file 1471-2148-6-92-S2.doc]

**Table S2. Positively selected sites selected by Bayes empirical Bayes analysis using the Fcodon and Fequal models (n=29)**

Sites for foreground lineage:

Fcodon model:

4Q 13R 17M 19T 22Q 26A 29C 40K 42Y 51W 53L 63T 69G 70S 71D 75Q 76L 78T 80Y 100Y 103G 112E 137S 142T 145Q 147S 163S 195G 198N 204E 215S 236E 238Q 244N (at *P* > 0.95)

Fequal model:

4Q 17M 19T 22Q 26A 51W 53L 63T 74L 75Q 76L 147S 163S 195G 198N 236E (at *P* > 0.95)
